# Letter from the Editor in Chief

**DOI:** 10.19102/icrm.2019.100303

**Published:** 2019-03-15

**Authors:** Moussa Mansour


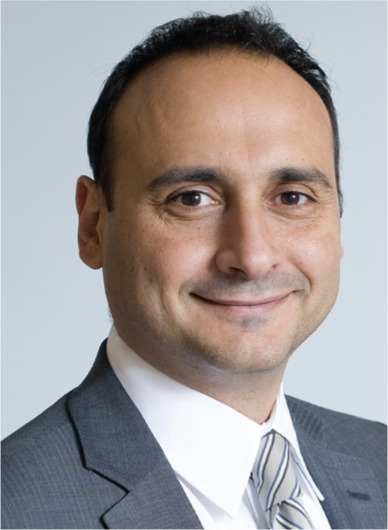


Dear Readers,

The volume of catheter ablation procedures performed for the treatment of cardiac arrhythmias has grown significantly in the past few years, and this growth is expected to continue for the foreseeable future. Most of the upwards trend, however, has occurred in the area of atrial fibrillation (AF), with significantly less of an increase happening in ventricular tachycardia (VT) ablation. It is estimated that, in the next few years, the compound annual growth rate for VT ablation volume will be approximately 7% versus a rate of 17% for AF. There are few reasons for this discrepancy; among them is that the development of new tools for AF ablation has outpaced that for VT ablation. As a result, VT ablation continues to be a more challenging procedure to perform.

One commonly used technique for VT ablation, activation mapping, necessitates the induction of the offending arrhythmia, which is often unstable and requires multiple shocks and which frequently results in hemodynamic compromise. As a result, substrate mapping ablation has been felt to be a more effective choice for VT ablation. However, there is no agreement yet to date on the most optimal strategy for substrate mapping and ablation.

This issue of *The Journal of Innovations in Cardiac Rhythm Management* contains a very interesting article by Kitamura et al.^1^ titled “Substrate Mapping and Ablation for Ventricular Tachycardia in Patients with Structural Heart Disease: How to Identify Ventricular Tachycardia Substrate.” In it, the authors discuss the challenges seen with different available techniques for VT ablation. These include local abnormal ventricular activity–guided ablation, linear ablation with cross-section of the scar and border-zone, scar homogenization, border-zone ablation/core isolation, and scar dechanneling. Of note, though, another promising option that the authors describe is scar definition by way of cardiac magnetic resonance imaging or computed tomography with integration of the resulting image with the electranatomical map acquired during the procedure. This technique appears to be promising because it allows for scar definition to occur in a noninvasive way prior to the procedure, including localization of the electrical isthmuses, which can significantly reduce the procedure time. This method of ablation is likely to facilitate improved ease and outcomes of VT ablation in a big way. However, for it to be fully effective, it must be coupled with the development of new ablation catheters specifically designed for VT ablation that allow for the creation of deeper lesions that those required in AF ablation, which hopefully we will see in the near future.

I hope that you enjoy reading this issue of *The Journal of Innovations in Cardiac Rhythm Management*.

Sincerely,


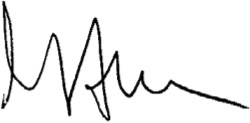


Moussa Mansour, md, fhrs, facc

Editor in Chief

The Journal of Innovations in Cardiac Rhythm Management

MMansour@InnovationsInCRM.com

Director, Atrial Fibrillation Program

Jeremy Ruskin and Dan Starks Endowed Chair in Cardiology

Massachusetts General Hospital

Boston, MA 02114

## References

[1] Kitamura T, Martin CA, Vlachos K (2019). Substrate mapping and ablation for ventricular tachycardia in patients with structural heart disease: how to identify ventricular tachycardia substrate.. J Innov Cardiac Rhythm Manage..

